# Detection of the dominant direction of information flow and feedback links in densely interconnected regulatory networks

**DOI:** 10.1186/1471-2105-9-424

**Published:** 2008-10-08

**Authors:** Iaroslav Ispolatov, Sergei Maslov

**Affiliations:** 1 Ariadne Inc., 9430 Key West Ave. Suite 113 Rockville, MD 20850, USA; 2 Departamento de Fisica, Universidad de Santiago de Chile, Casilla 302, Correo 2, Santiago, Chile; 3 Department of Condensed Matter Physics and Materials Science, Brookhaven National Laboratory, Upton, New York 11973, USA

## Abstract

**Background:**

Finding the dominant direction of flow of information in densely interconnected regulatory or signaling networks is required in many applications in computational biology and neuroscience. This is achieved by first identifying and removing links which close up feedback loops in the original network and hierarchically arranging nodes in the remaining network. In mathematical language this corresponds to a problem of making a graph acyclic by removing as few links as possible and thus altering the original graph in the least possible way. The exact solution of this problem requires enumeration of all cycles and combinations of removed links, which, as an NP-hard problem, is computationally prohibitive even for modest-size networks.

**Results:**

We introduce and compare two approximate numerical algorithms for solving this problem: the probabilistic one based on a simulated annealing of the hierarchical layout of the network which minimizes the number of "backward" links going from lower to higher hierarchical levels, and the deterministic, "greedy" algorithm that sequentially cuts the links that participate in the largest number of feedback cycles. We find that the annealing algorithm outperforms the deterministic one in terms of speed, memory requirement, and the actual number of removed links. To further improve a visual perception of the layout produced by the annealing algorithm, we perform an additional minimization of the length of hierarchical links while keeping the number of anti-hierarchical links at their minimum. The annealing algorithm is then tested on several examples of regulatory and signaling networks/pathways operating in human cells.

**Conclusion:**

The proposed annealing algorithm is powerful enough to performs often optimal layouts of protein networks in whole organisms, consisting of around ~10^4 ^nodes and ~10^5 ^links, while the applicability of the greedy algorithm is limited to individual pathways with ~100 vertices. The considered examples indicate that the annealing algorithm produce biologically meaningful layouts: The function of the most of the anti-hierarchical links is indeed to send a feedback signal to the upstream pathway elements. Source codes of *F*90 and Matlab implementation of the two algorithms are available at

## Background

During the last several years a substantial amount of information on large-scale structure of intracellular regulatory and signaling networks has been accumulated. However, the growth in our understanding of how these networks function in a robust and specific manner was lagging behind the shear rate of data acquisition.

To be able to understand the biological functioning or even to efficiently visualize a complex regulatory and signaling network it is important to determine the dominant direction of the information flow and to identify the links that go against this flow and thus generate feedback loops. Ordering a network in such as way that the information cascades down from higher to lower hierarchical levels can help to detect its previously unknown inputs and outputs, to track sources of perturbations based on their observable downstream effects, etc. A simple-minded hierarchical layout of a densely interconnected network is often impossible due to a ubiquitous presence of feedback loops. Indeed, all nodes in a strongly connected component of a network by definition are simultaneously upstream and downstream of each other.

However, if the forward flow of information in the network along multiple channels dominates over the backward flow along relatively few feedback links, the proper hierarchical layout could still be reconstructed based on the network topology alone. The identification and removal of a small number of feedback links would enable one to perform the hierarchical layout of the remaining acyclic network. In the next section we introduce a new probabilistic algorithm to detect an optimal hierarchical layout which minimizes the number of feedback links going from lower to higher levels in the hierarchy. In addition to direct biological applications, this algorithm provides a new computational approach to one of the 21 classic Karp's NP-hard problems: finding the Minimum Feedback Arc Set in a directed graph [1]. This problem enjoys a seemingly everlasting popularity reflected in a substantial number of approximate solutions (see, for example, [2-4] and references therein). It has also been shown that the Minimum Feedback Arc Set problem, apart from being NP-complete, is also APX-hard, which means that there exists a constant *k *> 1 (often called the approximation factor) such that there is no polynomial-time approximation algorithm that always finds a link set at most *k *times bigger than the optimal result. The first polynomial approximation algorithm for the feedback arc set problem was designed by Leighton and Rao [4,5] with the approximation factor *k *~ O(log^2 ^N) where *N *is the number of graph vertices. This estimate for the approximation factor was improved by Seymour to O(log N log [log N]) for polynomial-time algorithms.

Many of the existing approximate algorithms to the Minimal Arc Set problem are deterministic and greedy in nature, which on one hand side allows a fairly precise prediction of their complexity, performance and precision, yet on the other hand side involves consequences such intrinsic "near-sightedness" and frequent inability to find the global optimum among the local ones. A good example of such deterministic greedy algorithm which also relates the minimum feedback arc set search to graph layout is presented in [6]. According to this algorithm the hierarchical level of a node is determined by the difference between its out-and in-degrees. This way, the nodes with larger than average out-degrees and/or smaller than average in-degrees are naturally placed among the top hierarchical layers. These nodes directly or indirectly control other nodes with larger than average in-degree and/or smaller than average out-degree. The number of operations in this approximate algorithm scales linearly with the number of graph links.

Our proposed algorithm, unlike the existing algorithms described above, is probabilistic in nature. In the limit of sufficiently slow and long annealing it has a good chance to converge to the actual solution of the minimum feedback arc set problem. At the same time it still requires only a polynomial number of operations, which is proportional to the product of the number of links and vertices in the graph. To evaluate the advantages of the proposed annealing algorithm and to reveal its distinction from the deterministic algorithms, we compare it to our own greedy deterministic algorithm. This algorithm sequentially cuts the links that belong to the largest number of cycles in the network. We found that the probabilistic simulated annealing algorithm generally outperforms the deterministic one in both the number of removed feedback links (which needs to be minimized) as well as in the speed and memory requirements. A simple visual example is provided for the situation when the deterministic greedy algorithm is non-optimal.

Following that, we discuss biological implications and applications of our findings as well as how additional constraints such as *a priori *knowledge of the function and, therefore, the hierarchical position of certain nodes affects the resulting layout.

## Methods

Consider a graph consisting of *N *vertices and *L *directed links. The goal is to distribute the vertices among *M *levels in such a way that the number of links going against the hierarchy, or from a lower level to the same or a higher one, is at its minimum. If the number of levels *M *is sufficient (equal or larger than the longest simple graph path), this problem is equivalent to finding a Minimum Feedback Arc Set [1], or removing as few as possible links to make the graph acyclic, or feedback-free.

A naive way to solve this problem exactly is to enumerate all cycles in a graph and then sample all possible combinations of links checking if they belong to all cycles. If one starts with enumerating individual links, then pairs of links, etc, until a removal of *l *links would yield the first acyclic graph, such sampling would require checking the $\sum_{i=1}^{l}\left(\begin{array}{c} L\\ i \end{array}\right)$ combination of links. For biologically relevant values *L *~ 10^3 ^- 10^4 ^and *l *~ 10^2 ^- 10^3 ^the computational costs of such exhaustive enumeration are prohibitive. (From an obvious identity, $\sum_{i=1}^{L/2}=\left(\begin{array}{c} L\\ i \end{array}\right)$, it follows that even for fairly modest *L *= 10^2^and *l *= *L*/2 the number of such attempts is ~10^15^.)

### Simulated annealing algorithm

The task of finding the minimum number of anti-hierarchical or feedback links can be interpreted as an optimization problem and tackled by probabilistic methods such as simulated annealing. Evidently, there exist more than one way to define the optimization function, and after exploring several possibilities we converged to the following one:

• For a given network, a set of *M *levels is introduced (*M *≤ *N*, in reality, *M *≪ *N *and is of the order of the graph diameter). Initially, all nodes are distributed on the levels randomly.

• For a particular distribution of nodes on levels, the number of links *L*_*opposite *_that go opposite to the hierarchy, that is, from a lower level to the same or a higher one, is declared to be the energy *E *= *L*_*opposite *_of the distribution, or the optimization function.

• A node and its new level are selected at random. A difference in energy Δ*E *that would occur if the node were moved to the new level is calculated. The node is moved to this new level with the probability min{1, exp(-Δ*E*/*T*)}, where *T *is the temperature.

• After the network has been sampled a sufficient number of times (of the order of *N *× *M*) so that each node has an opportunity to be moved to every level, the temperature is reduced by some factor, usually 0.9. Initially, the temperature is set sufficiently high, usually of the order of the average node degree *L*/*N*, to allow un-obstructed level changes.

• When the temperature becomes low enough to inhibit any level changes, the remaining ascending and same-level links are declared feedbacks and removed.

• The whole procedure can be repeated several times to check for consistency in the assignment of feedback links and to determine the solution with the lowest number of removed links.

**Figure 1:**
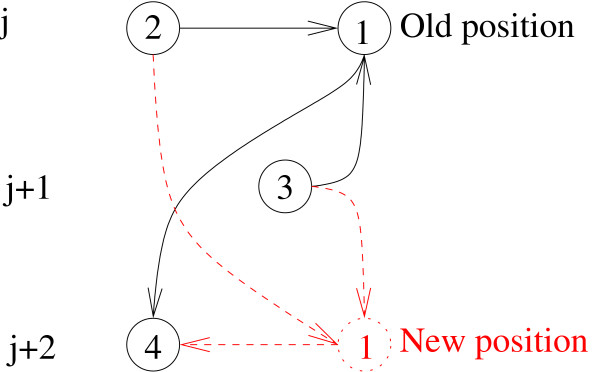
**Node 1 with two incoming and one outgoing links is selected to be moved from its current position on the level *j *to a new position on the level *j *+ 2.** The associated energy difference is Δ*E *= -1 - 1 + 1 = -1 where two -1 contributions come from making (2, 1) and (3, 1) links hierarchical and the single +1 contribution comes from turning the link (1, 4) from hierarchical to anti-hierarchical.

The energy difference associated with changing the level of a single node is illustrated in Fig. 1.

## Results and Discussion

### Comparison with deterministic greedy algorithm

To illustrate the advantages of the proposed simulated annealing method, we compare it to a straightforward "greedy" algorithm which follows the steepest descent in the number of feedback cycles. We implemented it in the following way:

• By enumerating all cycles in a graph, each link is assigned a score equal to the number of feedback cycles it participates in.

• The link with the highest score is removed. When several links have the same highest score, a link to be removed is selected among them by random.

• The score of each remaining link is reduced by the number of cycles which pass through this link and were cut on the previous step.

• The procedure of link removal and score reduction is repeated until no cycles remain (which means that scores of all links become zero).

The cycle enumeration can be implemented by following all paths that originate from a given vertex and recording only the cycles that come back to this vertex. The procedure is repeated for each of the *N *graph vertices: evidently, each cycle of length *C *is counted *C *times and a proper normalization is performed. Naturally, the performance of the greedy algorithm is limited in terms of speed and memory requirement of the cycle enumeration step.

**Figure 2:**
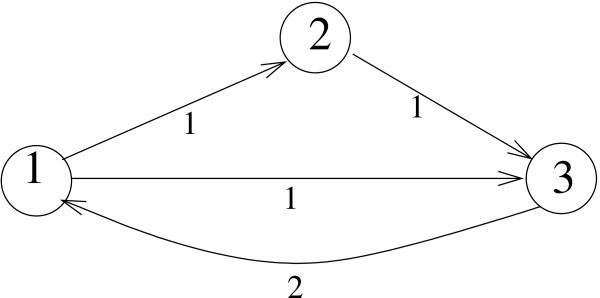
Removal of a single (3, 1) link makes this 3-vertex graph acyclic.

An example of network where the greedy algorithm performs flawlessly is shown in Figure 2.

Here the link (3, 1) carries the maximum score 2. A removal of this link indeed makes the graph acyclic, while a removal of any other than (3, 1) link would require a subsequent removal of the second link to achieve the same goal.

**Figure 3:**
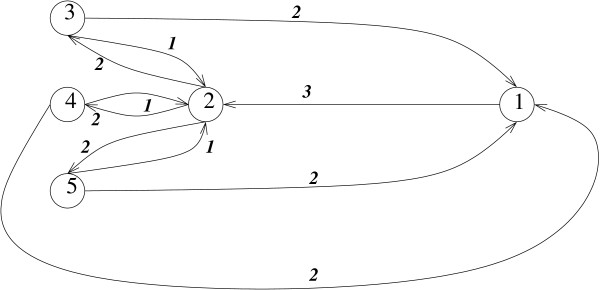
**An example of a network where the greedy algorithm fails to determine the optimal solution.** The initial link scores are shown. The link (1, 2) carries the highest score 3 and thus is cut first. However, three 2-node cycles {2, 3}, {2, 4}, and {2, 5} remain to be eliminated, after which the number of removed links becomes 4. The optimal solution would be to cut only three links (2, 3), (2, 4), and (2, 5), each carrying the score 2. This optimal solution has almost always been found by the annealing algorithm.

The hierarchical level-ordering by stimulated annealing outperforms the deterministic greedy algorithm in all respects. The performance of the stochastic stimulated annealing algorithm scales as *N *× *M*; memory-wise, it needs only lookup tables of a node position in the hierarchy and its nearest neighbors. Yet the greedy algorithm requires tracking along all paths originating from a given vertex, which uses a lot of memory and slows the performance significantly. Despite the fairly large prefactor required for a gradual multi-step annealing, the stochastic algorithm readily performs layouts of protein networks in whole organisms, consisting of around ~10^4 ^nodes and ~10^5 ^links. On contrary, we found it impractical to apply the greedy algorithm to networks with more than 100 – 200 vertices, which limits its utility to analyzing isolated systems and pathways. In addition, the greedy algorithm, as any other method based on near-sighted, local, single-step optimization, may miss the globally optimal solution, while the properly executed stimulated annealing always has a high probability of converging to it. This is indeed the case for bigger and more complex networks; yet even in a fairly simple graph, such as shown in Fig. 3, the greedy algorithm may perform non-optimally.

### Improving the layout by shortening the total length of hierarchical links

A good hierarchical layout of a regulatory or signaling pathway clarifies its biological functioning by identifying the cascade of information processing steps. Such layout should not only minimize the number of anti-hierarchical (feedback) links but also shorten the length of hierarchical (feedforward) links. Without carrying any energetic penalty these latter links can be arbitrarily long, i.e. they could connect proteins separated by many hierarchical levels. This interferes with identifying the hierarchical levels as definite stages (e.g. in a temporal sense) of information processing. Introduction of a small energetic penalty for the total length of hierarchical links alleviates this problem. The energy function used in our simulated annealing algorithm then becomes

(1) $$E=\sum_{i\to j}\left[H(m_{i}-m_{j})+(m_{j}-m_{i})E^{\prime }H(m_{j}-m_{i})\right].$$

Here *m*_*i *_and *m*_*j *_denote the hierarchical levels of nodes *i *and *j *connected by a link *i *→ *j *(or (i, j)) and *H*(*x*) is a version of the Heaviside step function equal to 1 for *x *≥ 0 and to 0 otherwise. For moderate-size pathways consisting of ~10^2 ^vertices we tried the range of energetic penalty *E*' = 0.02 - 0.1 (relative to 1, which is the energetic cost of a single anti-hierarchical link). The layout results in this range were independent of *E*'.

### Selecting the number of hierarchical layers

**Figure 4:**
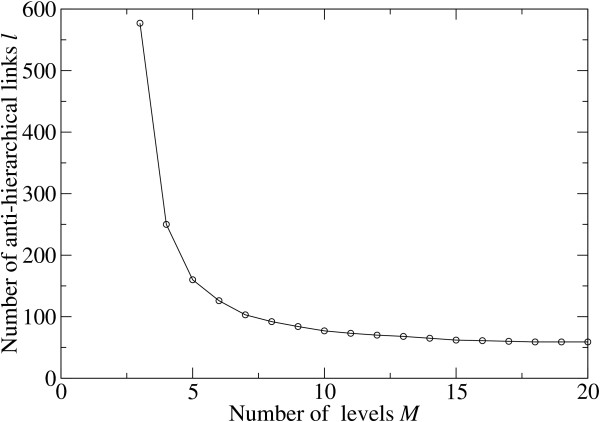
**The number *l *of anti-hierarchical links vsthe number of levels *M *in the annealing layout of the combined(a union of **[10]**and**[9]**datasets) protein phosphorylation network in human cell.** The network consists of *L *= 2880 links and *N *= 1297 nodes (proteins). The nodes with zero in-degree and zero out-degree are always put on the top and bottom levels, correspondingly. The leftmost data point corresponds to the single intermediate level (3 levels total), the number of anti-hierarchical links clearly reaches its minimum of 59 links for *M *≥ 18, which apparently is the length of the largest simple path in this network.

The number of levels *M *for the stimulated annealing hierarchical layout could be fixed by external biological requirements such as e.g. a limit on time allowed for a functional response. Otherwise, *M *could be determined self-consistently from our algorithm itself, by observing when the number of anti-hierarchical links stops to significantly decrease upon the increase in the number of levels. This is illustrated in Fig. 4 where a plot of the number of anti-hierarchical links vs number of levels is presented for the human protein phosphorylation network.

### Example: the layout of EGFR1 and B-cell receptor pathways

**Figure 5:**
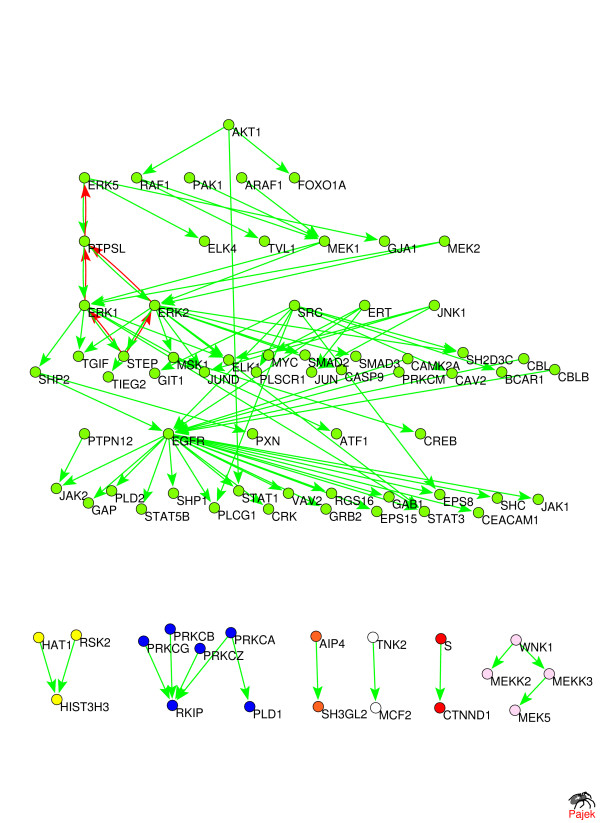
**Hierarchical layout of the EGFR1 pathway downloaded from the HPRD pathway database **[7]. The optimal layout was achieved at 7 hierarchical layers. Five predicted anti-hierarchical links are shown in red.

**Figure 6:**
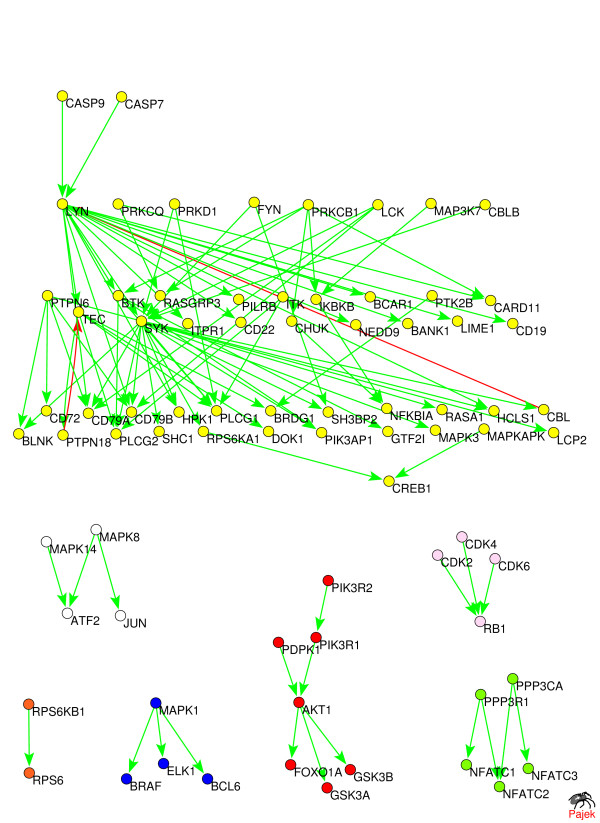
**Hierarchical layout of the B-cell receptor pathway downloaded from the   HPRD pathway database **[7]. The optimal layout was achieved at 5   hierarchical levels. Two predicted anti-hierarchical links are shown in red.

The biological function of anti-hierarchical links identified by our annealing algorithm often involves sending feedback late in the timecourse of their signaling pathways, or, similarly, resetting the pathway to its original state which it had before the arrival of the signal. To illustrate this on a concrete biological example, we performed the hierarchical layout of two of the largest pathways in the HPRD pathway database [7]: the EGFR1-pathway (80 proteins, 90 regulatory interactions) and the B-cell receptor pathway (77 proteins, 90 regulatory interactions). The hierarchical layout is illustrated in Fig. 5 and 6. The optimal layout was achieved at 7 hierarchical layers for the EGFR1-pathway and 5 hierarchical levels for the B-cell receptor pathway. In most annealing runs we correspondingly identified 5 and 2 anti-hierarchical links in these two pathways.

It is intriguing that 5 out of these 5 + 2 = 7 anti-hierarchical links correspond to dephosphorylation and 1 to ubiquitination protein modification processes. While these biomolecular mechanisms alone do not prove that the involvement of these interactions in feedback, they strongly support this notion. Indeed, dephosphorylation is commonly used to reset previously phosphorylated proteins to their original state, which they had before the arrival of the signal. Similar to this, the ubiquitination of a protein triggers its degradation by the proteasome, which once again resets the state of the pathway to what it was before the arrival of the signal. Thus protein modifications due to dephosphorylation and one to ubiquitination tend to happen late in the timecourse of signaling pathways and thus likely to be used for feedback signaling. In addition to these direct applications, our algorithm is useful, for example, for identifying putative sources (signaling inputs) of multigene differential expression patterns [8]. Such procedure is based on tracking upstream the regulatory links from often very numerous differentially expressed genes to the common regulators that could have caused the particular expression pattern. To be able to do this one needs a network of direct or indirect protein regulations from which all feedback links have been previously removed.

### Example: the layout of the genome-wide network of human post-translational modifications

**Figure 7:**
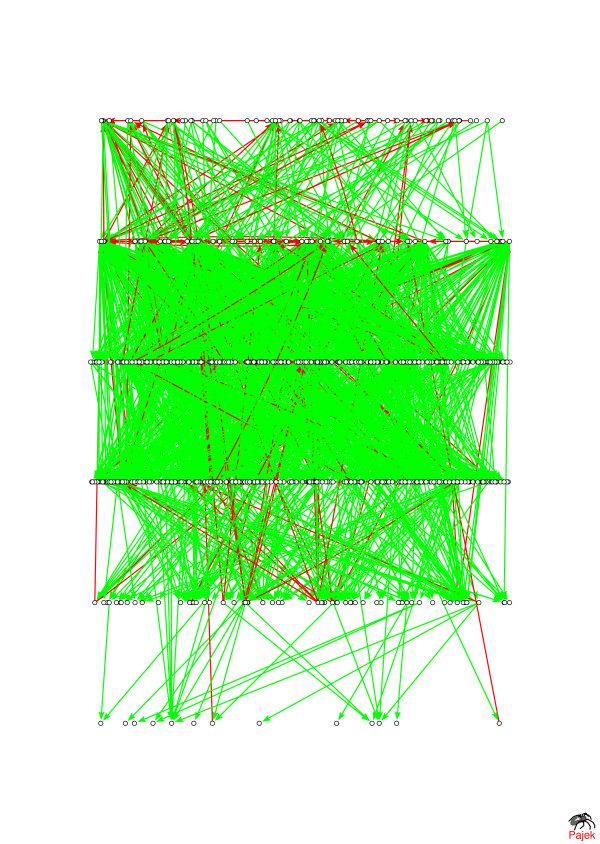
**The network**[9]**of post-translational modifications (phosphorylation/dephosphorylation, proteolytic cleavage, etc.) of human proteins shown here includes 1671 automatically and manually curated edges between 732 proteins.** listed in the ResNet 4.0 database. The hierarchical layout shown here is generated by our simulated annealing algorithm. Green arrows represent hierarchical links while red arrows – 208 anti-hierarchical (putative feedback) links going from lower to higher (or the same) levels in the hierarchy. Only proteins and links reachable from one of the 71 receptor-proteins placed at the top hierarchical level were included.

Often there exist some *a priori *biological knowledge about hierarchical positions of certain protein nodes in a signaling network. For example, the receptor proteins localized in the membrane typically serve as entry point of extracellular signals. Upon activation they pass these signals to cascades of proteins localized in the cytoplasm which ultimately reach the transcription factors localized in cell's nucleus. Thus receptor proteins might have to be forcefully put on the upper level of the hierarchical layout of a signaling network. We implemented this idea in the optimal layout of the network of post-translational modifications of human proteins shown in Fig. 7. This network is based on the ResNet 4.0 database [9], collected by Ariadne Genomics, Inc. from the biomedical literature with the help of Natural Language Processing algorithms. The information about protein-protein interactions was collected from abstracts of the entire PubMed database as well as full-text articles of more than 40 journals. It was then manually and automatically curated to include a reliable set of protein-modification interactions (phosphorylation/dephosphorylation, proteolytic cleavage, etc.) between human proteins. The optimal layout of 732 proteins in this network over six hierarchical levels is shown in Fig. 7. Green arrows represent 1453 hierarchical links while red arrows – 208 anti-hierarchical (putative feedback) links going from lower to higher (or the same) levels in the hierarchy. Only proteins and links reachable from one of the 71 receptor-proteins placed at the top hierarchical level were included.

In contrast to receptors, many transcription factors serve the role of effectors of signaling pathways and thus must occupy the lowest levels of the hierarchy. Initial positioning of such nodes at their appropriate hierarchical levels usually speeds up finding the layout with the smallest number of anti-hierarchical links. In addition, fixing these nodes on their appropriate hierarchical levels helps to find a layout which is more plausible from the biological standpoint.

In a similar way, the orientation of certain links (or unconnected pairs of proteins) could be fixed "by hand" if they are known to be of the feedforward or feedback nature. This could be implemented, for example, by assigning sufficiently large negative energies to the proper orientation of such links or protein pairs, making their annealing re-orientation highly improbable. Even imperfect (probabilistic) initial knowledge of biological functioning of the network could be used to assign weights to individual links, so that the energy *E *of a particular assignment of nodes to layers is a sum of weights of the anti-hierarchical links. Thus the *a priori *plausibility of a link to be (or not to be) a feedback can be introduced into the layering algorithm. We leave these questions as well as optimization of the proposed algorithms to various signaling and regulatory intracellular networks for future studies and publications.

## Conclusion

We introduced the simulated annealing algorithm, which is capable of performing near-optimal acyclic layout of large directed networks. It reveals the dominant direction of information flow in and identifies the set of links going against this dominant direction, that is from from lower to higher hierarchical levels. In biological regulatory and signaling networks such anti-hierarchical links often turn out to be involved in sending feedback or resetting signaling pathway to its default state. In addition to elucidating biological functioning of complex biomolecular pathways and networks, the proposed algorithm also offers a new probabilistic approach to one of the 21 classical NP-hard problem: finding the Minimum Feedback Arc Set in a directed graph.

## Authors' contributions

Both authours contributed equally to all parts of this work.
